# Overt Visual Attention as a Causal Factor of Perceptual Awareness

**DOI:** 10.1371/journal.pone.0022614

**Published:** 2011-07-25

**Authors:** Tim C. Kietzmann, Stephan Geuter, Peter König

**Affiliations:** 1 Institute of Cognitive Science, University of Osnabrück, Osnabrück, Germany; 2 Department of Systems Neuroscience, University Medical Center Hamburg-Eppendorf, Hamburg, Germany; University of British Columbia, United States of America

## Abstract

Our everyday conscious experience of the visual world is fundamentally shaped by the interaction of overt visual attention and object awareness. Although the principal impact of both components is undisputed, it is still unclear how they interact. Here we recorded eye-movements preceding and following conscious object recognition, collected during the free inspection of ambiguous and corresponding unambiguous stimuli. Using this paradigm, we demonstrate that fixations recorded prior to object awareness predict the later recognized object identity, and that subjects accumulate more evidence that is consistent with their later percept than for the alternative. The timing of reached awareness was verified by a reaction-time based correction method and also based on changes in pupil dilation. Control experiments, in which we manipulated the initial locus of visual attention, confirm a causal influence of overt attention on the subsequent result of object perception. The current study thus demonstrates that distinct patterns of overt attentional selection precede object awareness and thereby directly builds on recent electrophysiological findings suggesting two distinct neuronal mechanisms underlying the two phenomena. Our results emphasize the crucial importance of overt visual attention in the formation of our conscious experience of the visual world.

## Introduction

Conscious object recognition and overt visual attention belong to the most essential capabilities of the human visual system and cognition. Because both substantially contribute to our everyday experience of the world, they have moved into the center of scientific interest. Although there is evidence for their interconnection on a behavioral level [1], [2], the two phenomena were recently shown to rely on distinct neuronal mechanisms [3], [4], and are most often investigated in isolation [5], [6], [7], [8], [9]. As a result, the exact roles and temporal dynamics governing the interplay of the two processes remain unclear. In this context, one of the most fundamental questions is whether overt visual attention has a causal impact on the perceptual outcome of the recognition process (also named object perception hereafter), or whether the direction of causality is in fact reversed and that the awareness of an object's identity guides subsequent patterns of eye-movements.

These two views can be characterized by two hypotheses. The first hypothesis sees overt visual attention as following the perceptual outcome. According to this view, fixations are guided towards crucial local features of the object only after the subjects are aware of its identity (*action follows perception* hypothesis) [10]. The competing hypothesis suggests that features that are attended to prior to recognizing the object substantially contribute to the perceptual outcome (*action precedes perception* hypothesis) [11]. In this scenario, fixation patterns are in line with the upcoming percept prior to the actual awareness of the object identity.

**Figure 1:**
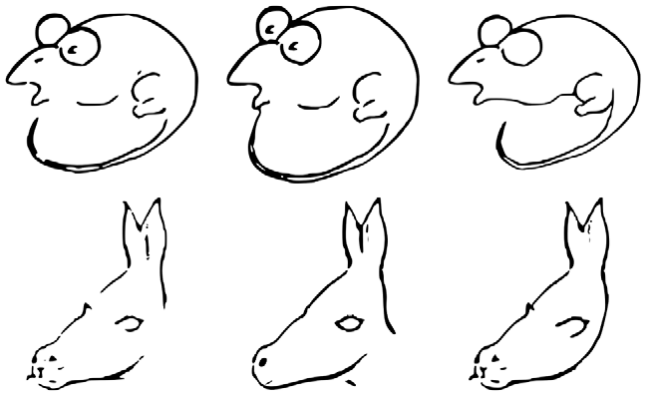
Exemplary Stimuli. Two out of the 12 stimulus sets used in the experiment. Each row shows an ambiguous image in the first column with the two unambiguous versions next to it.

To probe these two hypotheses, we conducted two eye-tracking experiments based on a set of twelve ambiguous stimuli. In order to provide a baseline of viewing behavior corresponding to the different perceptual outcomes, two unambiguous stimuli were created from every ambiguous one that bias the initial perception towards one of the two interpretations (Figure 1).

In the main experiment, experiment 1, we first investigate whether distinct patterns of overt visual attention precede different perceptual outcomes during the presentation of ambiguous stimuli. To ensure that only fixations prior to object perception are taken into account, we apply two correction methods. The first is based on the minimum reaction time of individual subjects during the complete experiment. The second is based on percept-related changes in pupil dilation that were recently shown to significantly precede perceptual events [12], [13]. Following this, we explore the possibility that object awareness has an effect on the subsequent patterns of overt visual attention. This is accomplished by comparing the viewing behavior of the subjects before and after the perceptual event.

In experiment 2, we investigate whether changes in the initial locus of visual attention, induced by shifting the initial gaze position of the subjects to different parts of the stimulus, would have a causal effect on the later perceived object identity.

## Experiment 1

### Experiment 1: Materials and Methods

#### Participants

Seventy-eight subjects, recruited via university mailing lists, took part in the experiment. The data of five subjects was discarded due to insufficient calibration accuracy. Forty-nine of the remaining 73 subjects were female. All subjects had normal or corrected to normal visual acuity and were informed of their right to withdraw from the experiment at any time without the need to state a reason and gave written informed consent to participate. Furthermore, all subjects were informed of the experimental procedure and were naive to the purpose of the study. Upon completion of the overall experiment, the subjects were debriefed. The study, including experiments 1 and 2, was approved by the Ethics Committee of the University of Osnabrück.

#### Stimuli

The used stimulus set consisted of 12 ambiguous stimuli, for each of which two additional disambiguated versions were created (leading to 36 stimuli in total). To create the disambiguated versions, the ambiguous stimuli were altered such that they would favor either one of the two percepts (see Figure 1 for an example). This was accomplished by manually adding or deleting small elements of the original ambiguous images. The 12 ambiguous stimuli included a version of the ambiguous donkey/seal figure [14], an image allowing for the percept of a woman's face or a saxophone player by Sara Nader, the man/mouse figure [15], an ambiguous stimulus showing a duck and a rabbit [16], the squirrel/swan figure of G. H. Fischer, “My Wife and Mother-in-law” [17], “My Husband and Father-in-law” [18], an instance of the images used in Fisher's hysteresis experiments [19], an ambiguous image showing either a couple or a rose, and, finally, an image showing a hand and a dancer. Included, but not used for analyses because all subjects reported the same initial percept of the ambiguous stimulus versions, was an image showing either a fist or a mother with her child, and a two- interpretation version of the Fisher family [20]. The complete set of stimuli is presented in Figure S1.

The stimulus presentations were interleaved with 36 black and white filler images showing animals and everyday objects. Each subject saw all 72 stimuli during the course of the experiment.

#### Apparatus

Eye tracking data were recorded using an Eyelink II system (SR Research Ltd., Mississauga, Ontario, Canada). It is capable of tracking both eyes; however, only the eye that gave a lower validation error after calibration was recorded at 500 Hz. No headrest was used. Stimulus presentation and response logging were programmed in python. For stimulus presentation, we used a 30-inch Apple Cinema Display (Apple Inc., Cupertino, CA, USA) with a native resolution of 2560×1600 px and an average response time of 14 ms. The stimuli, which were scaled to a size of 1000×1000 px, were presented centrally and in front of a white background. The distance to the screen was 60 cm such that the stimuli covered approximately 23.8° of the subject's visual field.

#### Task and Procedure

Subjects were individually tested in a dimly lit eye-tracking laboratory. After filling out a standard demographic questionnaire, the subjects were verbally introduced to the experimental procedure and by on-screen instructions. After successfully completing the calibration procedure (defined by reaching a validation error below 0.3°), the experiment was started. If required, the system was re-calibrated during the experiment.

Each trial started with a drift correction, requiring subjects to fixate the screen center. After manual acceptance by the experimenter, the stimulus appeared. Subjects were asked to press a response button as soon as they recognized the identity of the shown object. Following the button-press, the stimuli stayed visible for 4 more seconds. Although subjects were not explicitly informed of the potential ambiguity of the stimuli, they were asked to indicate changes in perception through additional button presses. After each stimulus had disappeared, subjects were asked to verbally report the perceived identity of the object. If multiple interpretations were reported, they were asked to give their reports in chronological order. Since the main interest of this study is an investigation of naïve, initial perceptual processing, the subjects were then asked to report whether they had ever seen the stimulus prior to the experiment.

The randomization of stimuli was accomplished as follows. Each subject saw one version of each stimulus during the first 24 trials. The presented stimuli contained four ambiguous and eight disambiguated stimulus versions, interleaved with a total of 12 fillers. The order of stimulus appearance was pseudo-randomized and counterbalanced across groups of four subjects out of which two were presented with the ambiguous version, one saw the disambiguated version A and one saw version B. This procedure was implemented in order to yield approximately the same amount of data for the ambiguous stimuli, which allowed for two distinct interpretations, as well as the unambiguous versions of the images.

#### Data Pre-Processing

Due to the objective of the current experiment, the data pre-processing procedure was rather restrictive. First, we discarded ambiguous-stimulus trials in which the reported percept did not match one of the two interpretations. For the unambiguous-stimuli, we excluded the trials in which the perceptual outcome was inconsistent with the intended interpretation (more than 80% of the percepts on the disambiguated stimuli were as intended, illustrating the efficacy of the stimulus manipulations). Additionally, trials in which the subjects indicated prior knowledge of the presented stimulus were excluded. Also trials that were either interrupted by the experimenter (no response after 20 seconds) (2,4%) or whose corresponding button press was outside the range of two standard deviations around the mean, were discarded (5,2%). After these steps, a set of n = 470 trials was left for further analyses.

In order to be able to investigate fixation behavior during the time of initial percept formation in a non-oscillatory setting, only fixations prior to subjects' object perception (i.e. the awareness of the perceived object identity) were selected for further analyses. For this, the subject's button press marks the upper limit of the time window of interest, because it also includes response preparation and execution in addition to the perceptual process. To exclude these response-related components, we identified the individual minimum reaction time (RT) across all trials for each subject and subtracted these minimum RTs from the recorded button press time points. Only fixations starting prior to this RT correction were used in further analyses. This method is quite conservative, as the correction estimate includes both, perceptual processing and the time required for the motor response of the shortest trial. Using this method, the fixation dataset was reduced by 28.6% (average minimum reaction time across subjects was 645 ms). As a control, we applied a second cleaning procedure based on changes in pupil dilation. As previously demonstrated in the literature [12], [13], the average pupil diameter significantly deviates from baseline prior to the perceptual reports. Using the pupil dilation method of fixation selection, for which dilation changes upon initial object recognition were compared to data collected in a follow-up experiment in which the subjects were asked to freely push a button without visual stimulation (see *Analyses S1 and Figure S3* for more details), the estimate of the average time ascribed to the motor-response was 528 ms prior to the button-press. Using this method, 26.1% of the data are discarded, rendering it less conservative than the RT-based approach. Because of this and because a subject individual procedure is clearly preferable to an experiment-wide cut-off, the following analyses were based on the RT method.

Finally, on the level of individual fixations, single data points that had no overlap with any others in a range of one degree of visual angle were treated as noise (2.1% of the fixations).

### Experiment 1: Analyses

The following analyses are based entirely on data collected during the subjects' first encounter with the experimental stimuli. As basis for analyzing viewing behavior, the recorded fixation data were first transformed into fixation density maps (FDM). These maps are created by first calculating 2D histograms of fixations across the stimuli, followed by a convolution with a Gaussian Kernel equivalent to 1° of visual field (FWHM = 42 pixels). The resulting maps were smoothed and normalized to a sum of one. For every set of stimuli (containing the ambiguous an the two disambiguated stimuli), FDMs were created for: ambiguous stimulus-percept A, ambiguous stimulus-percept B, disambiguated stimulus version A-percept A, disambiguated stimulus version B-percept B.

#### Comparing Viewing Behavior in Different Conditions

As a first analysis step, we compared the FDM's from the two disambiguated conditions against each other as well as the two perceptual conditions of the ambiguous stimuli. For this, we used a symmetric extension of the Kulback-Leibler (KL) divergence as a difference metric:
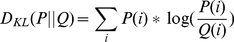
(1)

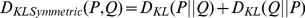
(2)To assess statistical significance of the found differences between FDMs we applied a separate bootstrapping analysis for each of the twelve stimulus sets. Using KL divergence as the test statistic, all subjects belonging to the two conditions to be compared were first pooled into one combined set. Resampling was then performed on the level of subjects, as resampling of individual fixations would violate the independence assumption of the bootstrapping analysis. In detail, two new sets of subject-data were randomly drawn with replacement from the overall set. It was ensured that the novel sets were identical in size, compared to the original ones. The resulting data was then used to calculate two new fixation density maps, for which the KL divergence was computed. The repetition of this procedure for 5000 times then leads to a distribution of KL divergences. This distribution describes the divergences that can be expected by chance, given the data. It can therefore be used as statistical distribution to which the original KL divergence can be compared. If the KL value of the original data falls into the highest 5% of values in this distribution, the null hypothesis of equal distributions can be rejected. To analyze statistical significance on the group level including all tested stimuli, the distribution of calculated p-values was then tested for uniformity (H_0_). If the FDMs from two conditions do not differ across all stimulus sets, a uniform distribution of p-values would be expected.

#### Predicting Perceptual Outcomes from Single Fixations

To assess whether it is possible to predict the later perceptual outcome of our subjects based on single fixations made prior to recognition, we trained stimulus-individual Support Vector Machines using the SVMlight implementation [21]. For each ambiguous stimulus, the raw fixation coordinates prior to recognition were used as input space. Prediction performance was evaluated with a leave-one-subject-out cross validation, i.e. the individual fixations of each subject were once excluded from training and used as test set for the classifier. Prediction performance was then assessed based on the average accuracy gained from classifying single fixations of the test subject. Averaged across subjects, we then yield the stimulus-individual prediction performance. Finally, the grand total predictability of the perception of the subjects based on singular fixations made prior to the actual recognition is obtained via subsequent averaging across the stimulus performances.

#### Alignment of Viewing Behavior on Ambiguous and Unambiguous Stimuli

In order to examine whether equal perceptual outcomes on the ambiguous and the corresponding unambiguous stimuli would be preceded by similar viewing behavior, a similarity index δ was defined. It is positive if the differences in viewing behavior between the ambiguous stimuli with different percepts are in the same direction as the differences on the unambiguous stimuli. δ is computed as follows. First, a difference map (D) is created for each stimulus set by subtracting the two unambiguous fixation density maps from each other. Then, the cosines of the angles between this difference map and the fixation density maps of all four conditions, one for each unambiguous stimulus (uFDM_A/B_) and one for each possible percept on the ambiguous stimulus (aFDM_A/B_), are calculated. The final similarity index (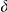), is then defined as the quotient between the difference of ambiguous cosines and the difference between the corresponding unambiguous cosines:
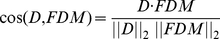
(3)

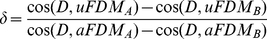
(4)Eq. (4) therefore expresses the differences between ambiguous conditions as fraction of the maximal possible difference, as estimated from the unambiguous reference FDMs. For the statistical analysis of the similarity index, a randomization analysis was performed. The approach was similar to the described bootstrapping in case of the KL divergence, but resampling was done without replacement and the index 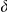 was used as a test statistic. As before, the original 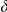-value was compared to the distribution of resampled 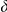 -values to obtain the p-value.

#### Temporal Analysis

Based on the similarity index 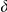, it is possible to analyze the temporal development of viewing behavior on the ambiguous stimuli with regard to different perceptual outcomes. For this, we first aligned the fixation data to the button press. Starting at the button press, we then shifted a time window of 200 ms backwards over the fixation data of each image set. Based on the fixations that fell into this time window, the similarity index was calculated. The final curve was calculated by averaging the *_δ_*-indices of all image sets.

#### Subject Level Analysis of Individually Collected Evidence

The previously described analyses were performed across subjects and therefore on the level of stimuli. However, by using the difference maps (D) created from the FDMs of the unambiguous stimuli as reference, it is also possible to investigate subject individual scan paths on the ambiguous images to check whether the subjects collected more evidence consistent with their later percept than for the alternative one. For this, the difference map is interpreted as depicting evidence for the different perceptual outcomes. Positive values in the difference maps represent evidence for percept A, whereas negative values correspond to evidence associated with percept B. To obtain an estimate of the individually collected evidence of a subject, the recorded scan-path on the corresponding ambiguous stimulus is projected onto the difference map. For each fixation along the trajectory, the collected evidence corresponds to the average values of the difference map within a circular region of two degrees of visual angle. The overall evidence of a scan path is then defined as the sum of evidence across all fixations. This value is positive, if the subject collected more evidence for interpretation A, whereas it is negative, if more evidence was collected for B.

To statistically evaluate whether subjects collected more evidence for their actual rather than the alternative interpretation of the ambiguous image, the individually collected evidence was sorted into two sets, according to the initial perception of the subjects. These sets were then tested with a Mann-Whitney U-test.

### Experiment 1: Results

Because our data pre-processing procedure is related to the reaction times of the subjects, these were analyzed first. We found that the unambiguous stimuli were significantly faster recognized than on ambiguous ones (median RT_unambiguous_ = 1.51 s±0.15 s.e.m., RT_ambiguous_ = 1.78 s±0.09 s.e.m.; s.e.m. will be used for each ± hereafter, Mann Whitney U-test *Z* = 3.46, p<0.001). Moreover, the average minimum reaction time across subjects was found to be 645 ms. After performing the described pre-processing procedure, excluding fixations that were made during the time window associated with the motor response, on average 3 fixations (distribution median) were left for further analyses in the unambiguous case and 4 fixations for the ambiguous stimuli.

#### Viewing behavior prior to object awareness

**Figure 2:**
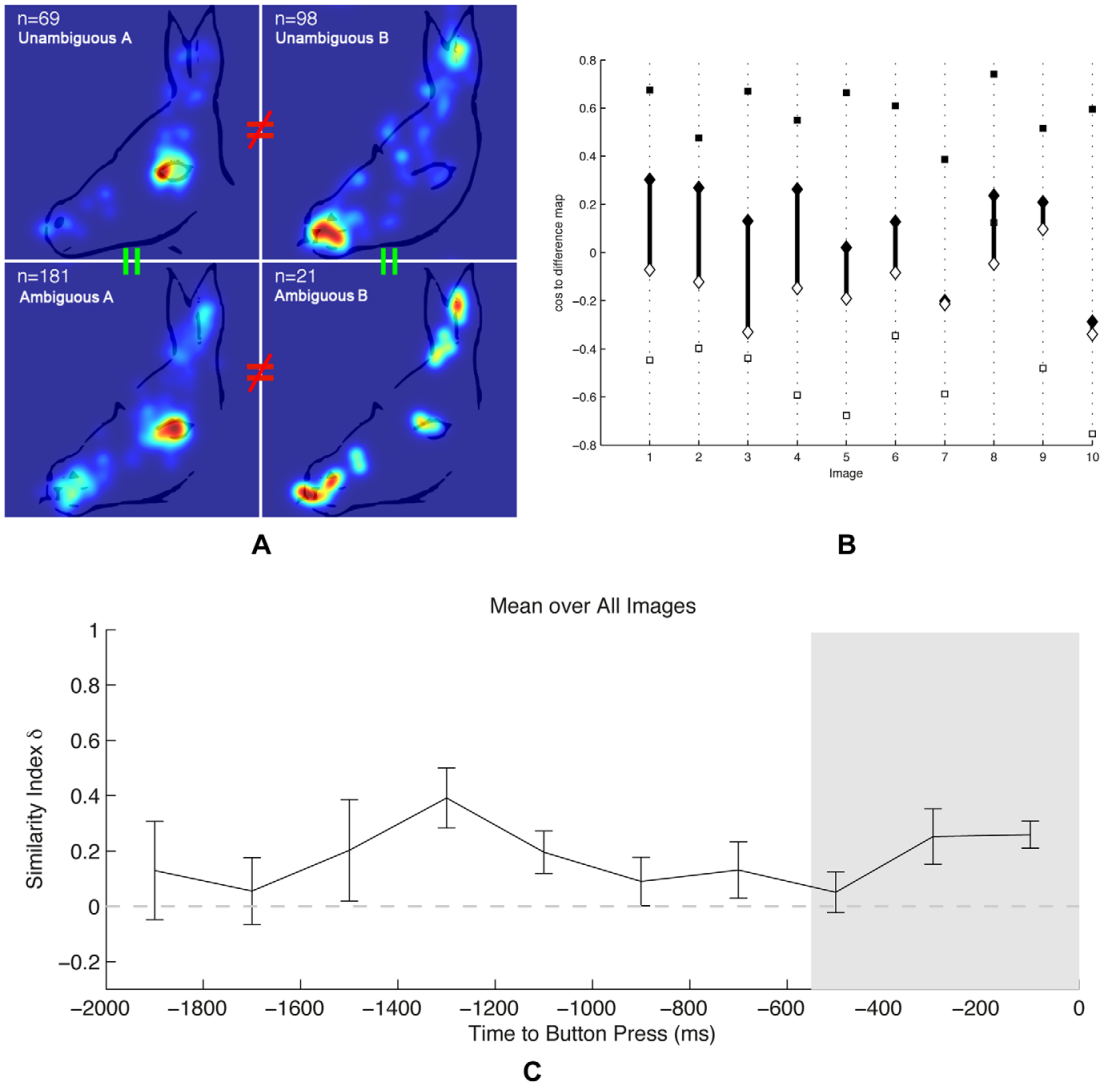
Viewing behavior prior to awareness. (**a**) Examples of viewing behavior prior to object awareness on the ambiguous and unambiguous stimuli with corresponding percepts. There are significant differences between the groups with different percepts (as indicated by the KL divergence analysis), and the differences in the viewing behavior on the ambiguous and unambiguous stimuli are aligned with identical percepts (as shown by the similarity index δ). The shown fixation patterns correspond to the fifth-largest index value out of the ten examined stimulus sets. (**b**) The cosine values underlying the similarity index calculation for the individual fixation density maps (FDM). Filled symbols represent percept A, the empty ones percept B. Squares denote cosines calculated from the unambiguous FDMs; diamonds indicate values calculated from the ambiguous FDMs. Image 1 corresponds to the example shown in (a). (**c**) The time-analysis showing the index-peak at about 1330 ms before the button press. Error bars are s.e.m. The shaded area marks the time during which data would be discarded according to the pupil analysis.

**Figure 3:**
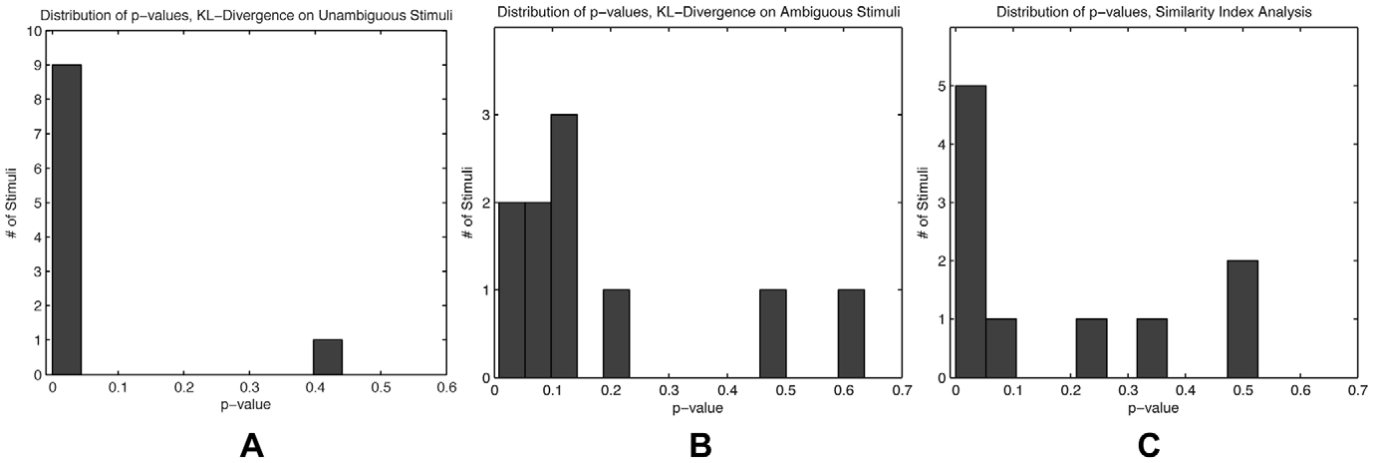
Bootstrapping Distributions. Shown are the distributions of p-values for (**a**) KL-Divergence on the unambiguous stimuli, (**b**) KL-Divergence on Ambiguous Stimuli with different percepts, (**c**) the similarity index δ. All of them are nonuniform and right-skewed.

As a first analysis of overt visual attention during the time in which no conscious recognition has yet occurred, we assessed whether differences in fixation patterns existed on sets of two unambiguous stimuli corresponding to the two interpretations of an ambiguous one. For this, we computed fixation density maps and used the described symmetric extension of the Kullback-Leibler (KL) divergence as difference metric. Bootstrapping revealed that the viewing behavior on the different unambiguous versions of the stimuli differed strongly and significantly across the stimulus set (see Figure 2a for an example). The p-values of the 10 stimulus sets obtained via bootstrapping are nonuniformly distributed, contrary to the tested null hypothesis of similar viewing behavior, and right- skewed (Chi^2^ = 10, p<0.01; Figure 3). Nine out of the ten analyzed stimulus sets are individually significant (p<0.01, Bootstrapping). In view of the subtle changes in the images, the robust differences in viewing behavior are remarkable. Furthermore, they form an important reference for the differences in viewing behavior that are to be investigated on the ambiguous stimuli.

**Figure 4:**
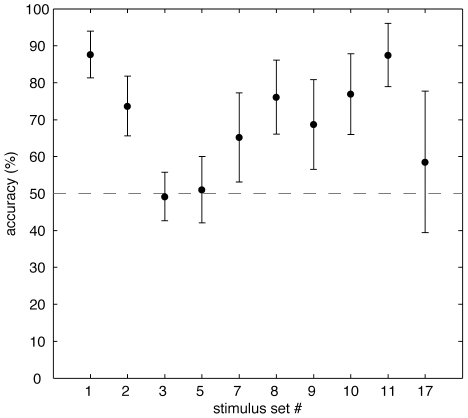
SVM performance. Mean Support Vector Machine prediction accuracy for the correct percepts is shown for the ten tested image sets. Accuracy over fixations of one subject was calculated using SVM's trained on the remaining fixation data of the ambiguous stimuli (leave-one-out cross validation). Errorbars depict SEM's.

The most important aspect with regard to the two examined hypotheses, *action follows perception* and *action precedes perception*, is the viewing behavior on the ambiguous stimuli. For this comparison, we grouped the fixation data according to the subjects' perceptual decisions. A comparison of the corresponding fixation density maps revealed that even in the case of an identical stimulus initial viewing behavior recorded prior to object awareness differed significantly for different perceptual outcomes. The distribution of p-values from the KL divergence bootstrapping is nonuniform and right-skewed (Chi^2^ = 6.4, p<0.025). Two stimulus sets were individually significant (p<0.01) (Figure 2a shows an example, the distribution of p-values can be seen in Figure 3). This is a particularly strong case, because only the perceptual formation process and the sampling of stimulus properties, but no differences in the presented stimuli can be associated with the found differences in overt attention. This finding implies that it should be possible to predict the perceptual outcome of our subjects based on their overt visual attention recorded prior conscious recognition. Put differently, it should be possible to predict the subjects' perception based on data that is recorded at a time in which the subjects themselves are not yet aware of their later percept. Indeed, after training radial-basis Support-Vector-Machines on the ambiguous fixations using a leave-one-out cross validation scheme, it was possible to predict the subjective percept with an average accuracy of 70% (±4%). Figure 4 shows the prediction accuracy for the individual stimuli.

Following the individual analysis of conditions based either on unambiguous or ambiguous stimuli, we assessed whether the viewing behavior on the ambiguous stimuli was aligned with that on the unambiguous ones upon similar perceptual outcomes. This step is crucial because it implies that the found differences on the ambiguous stimuli are in fact percept-related and not incidental. To assess the similarity, an index δ was defined by projecting fixations on the ambiguous stimuli onto the axis spanned by the differences of viewing behavior on the unambiguous stimuli (see *Methods* for more details). The index δ is positive if the differences in viewing behavior between the ambiguous stimuli with different percepts are in the same direction as the differences on the unambiguous stimuli. In all stimulus sets, the index was found to be positive (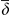 = 0.26±0.05; see Figure 3b), indicating that overt visual attention on the ambiguous stimuli was aligned with fixations on the corresponding unambiguous ones prior to conscious recognition of the shown object. A randomization analysis, analogously to the above Bootstrapping analyses, confirmed the statistical significance of the effect (see Figure 2; Chi^2^ = 6.4, p<0.025; five stimulus sets individually significant with p<0.05).

Our previous analyses were based on collapsed data taken from the complete period prior to conscious recognition. To analyze the temporal dynamics of overt visual attention in more detail, we performed a sliding window analysis. After aligning the trials to the button press and selecting data according to the current time-window, the mean similarity index δ exhibits a clear peak at about 1300 ms prior to button press (Figure 3c). At this time, which is largely before the later report of conscious recognition, the fixation behavior on the ambiguous stimuli is most similar to the corresponding unambiguous ones, and therefore most different for different percepts on the same ambiguous stimuli.

To approach the differences in viewing behavior on a subject instead of stimulus basis, the difference maps can be interpreted as depicting evidence for the different percepts. Now, each individual scan-path on an ambiguous stimulus represents subsequent collecting of evidence for one or the other percept, depending on whether a positive or negative region in the difference map is fixated. This analysis revealed that the evidence collected by subjects prior to recognition significantly differs for subjects with different percepts (median 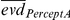 = 0.026±0.007, 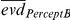 = −0.025±0.007, Mann Whitney U-test *Z* = 6.23, p = 4.7*10^−10^). As the sign of these two numbers shows, subjects collected more evidence for their actual than for the competing, but not perceived, percept.

#### Viewing behavior after a formed percept

By showing that significant and percept-aligned differences in pre-conscious viewing behavior exist on ambiguous stimuli, our results strongly favor the *action precedes perception* hypothesis. However, the results presented so far do not exclude the possibility that also object awareness had an effect on the subsequent overt visual attention. Since the stimuli were shown for four more seconds after the first button press of the subjects, it is possible to test this issue by comparing the viewing behavior before and after the reaction-time corrected perceptual report.

First, we tested only subjects, which did not report a change in perception during the 4-second period. Contrary to the predictions of the *action follows perception* hypothesis, we did not find evidence for an increase in probability to fixate characteristic local features as determined by the similarity index δ (t (9) = 2.1, p>0.05; normality and homoscedasticity verified by Lilliefors tests (p>0.05), and Bartlett's test (p>0.05)). Following this, we analyzed the data of the subjects who identified a second interpretation and therefore a switched perception. For this type of event, the *action precedes perception* hypothesis predicts a drop in similarity index prior to the perceptual switch, since subjects are expected to sample more evidence for the competing percept prior to becoming aware of the alternative interpretation. This test requires subjects who switch during ambiguous trials from an initial percept A to percept B and vice versa for each stimulus set. Despite the large number of subjects, the required data existed only for two ambiguous stimuli (stimulus 2 and 3) and therefore the sample size is not sufficient for detailed statistical analysis. However, we found a correct tendency, as the subjects with a perceptual switch exhibit a smaller index than the non-switching ones during the time between the two button presses (median*_image2,3_* 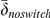 = 0.27, 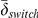 = 0.01).

Given the results of experiment 1, which illustrated significant differences in overt visual attention prior to conscious recognition, we investigated in a second experiment whether the found correlative relationship also has causal capacities. The experimental reasoning was that if the initially attended information has a causal effect, it should be possible to manipulate the perceptual outcome by means of changing the initial fixation of the subjects. Leaving everything else equal to the first study, we therefore manipulated the position of the fixation cross shown prior to stimulus presentation, and tested whether this would result in a changed perception of our subjects.

## Experiment 2

### Experiment 2: Materials and Methods

#### Participants

Twenty-six subjects (19 female) took part in the second experiment. None of them had participated in experiment 1. As before, the subjects received either 5€ or course credit for their participation. Six additional subjects took part in a pre-experiment to assess the altered positioning of the fixation cross.

#### Stimuli

In the second experiment we used the 10 ambiguous stimuli also included in the analyses of experiment 1 and ten of the previously used fillers.

#### Apparatus

The used apparatus and experimental setup was identical to experiment 1.

#### Task and Procedure

The experimental procedure of the second experiment was largely identical to the first. However, to check for the effect of visual attention on the later percept, we introduced an experimental manipulation in which the position of the initial fixation cross, shown before stimulus onset, was altered. For this, the new starting positions were shifted to locations that were expected to be consistent with either one of the two percepts. To determine these positions, six additional subjects that were not involved in any of the eye-tracking experiments, were asked to freely mark the regions of the ambiguous stimuli that “clearly favor one or the other percept”. Contrary to the main experiments, these subjects were informed about the two percepts beforehand in order to verify that each subject was able to perceive both versions. From the marked regions of these subjects, clusters with more than 80% congruence across subjects were selected and the cluster centroids were calculated. Following this, a straight line was drawn through both centroids. The new initial fixation points were positioned on this line at 1–1.5 degree of visual angle away from the centroids towards the image borders (see Figure S2).

Importantly, the introduced manipulation only changed the subjects' initial locus of visual attention as the fixation cross disappeared with stimulus onset and subjects were then allowed to freely move their eyes. Compared to earlier studies, which either forced the subjects' view onto a specified position during the complete trial [6], or which directly manipulated the stimuli in order to bias the perception towards one or the other outcome [22], this manipulation is very subtle and allows for very natural viewing behavior on the stimuli.

The starting position was balanced across subjects. Half of the subjects started at the location favoring interpretation A and the other half at position relevant for B. An experimental session lasted approximately 30 minutes.

#### Data Pre-Processing

Again, we excluded trials for which the subjects had indicated prior knowledge of the presented stimulus. Moreover, trials for which the reaction times fell outside of a 2 standard deviation range around the mean were excluded. On stimulus level, we excluded stimulus 6 (old/young woman), as all of the recorded subjects reported prior knowledge. Finally, we excluded stimulus 8 (man/woman taken from Fisher's hysteresis experiments) as an outlier because the results of the manipulation were more than two standard deviations away from the group average.

### Experiment 2: Results

We statistically assessed the efficacy of the manipulation based on two methods. First, we performed a Chi^2^ cross-tab test and found a significant dependence of the reported percepts on the initial fixation position (Chi^2^ = 5.74, p = 0.006). On average, 60.3% of the percepts were consistent with the bias induced by the starting position. Importantly, any perceptual biases in the ambiguous stimuli during the first experiment cannot explain this result, as their effect equally affects the result positively for one percept, but negatively for the other. Still, to explicitly account for the found biases of the stimuli in the original experiment, we performed a second analysis. For this, we first calculated the percentage of subjects perceiving A and B for every ambiguous stimulus in experiment 1. Then, we calculated the percentage of subjects who correctly perceived A (and B) in condition A (and B) during experiment 2 and calculated the percentage gained through the experimental manipulation on each stimulus by averaging the subtracted the percentages of experiment 1 from the percentages of experiment 2. As an example, if for a given stimulus in the original experiment A was perceived in 60% and B in 40% of the cases, and in experiment 2, 70% of the subjects perceived A in condition A and 60% perceived B in condition B, then the average percentage gain for this stimulus would be 15%. Once this was calculated for every stimulus, we checked the resulting distribution for a deviation from zero using a t-test (the normality assumption was verified with a Lilliefors test). The test showed that the introduced changes in the initial fixation positions had a significant effect on the perceptual outcome of the subjects (t = 3.45; p = 0.01).

This robust effect, which is in line with earlier studies emphasizing the importance of local features in fixed eye-position setups [6], [7], [16], [23], is quite remarkable because the subjects were allowed to freely move their eyes as soon as the stimulus appeared.

## General Discussion

The current work aimed at a clarification of the interplay between overt visual attention and object perception. We approached this problem by investigating patterns of viewing behavior preceding the conscious recognition of ambiguous stimuli and show that different percepts (and perceptual switches) are preceded by significant and percept-aligned differences in viewing behavior. In line with this, we demonstrated that eye-movements recorded prior to the conscious recognition are a good predictor for the later perceptual outcome, and that subjects collect more evidence for the later perceived object identity than for the alternative one. In experiment 2, we extended the correlative results from experiment 1 by showing that manipulations of the initially attended positions significantly influence the later perceptual outcome. This finding further clarifies the role of overt visual attention by providing evidence for a causal influence on perception. All of these results are completely compatible with the view that the object awareness follows overt visual attention (*action precedes perception* hypothesis). However, as the results do not exclude the possibility that also the awareness of object identity has an impact on the subsequent overt visual attention, we additionally compared the viewing behavior preceding and following object awareness. No significant difference in the similarity index could be found. Directly related to this, the interplay of hippocampus-dependent memory, in form of awareness of image manipulations, and patterns of overt visual attention were recently investigated [24], [25]. The authors conclude, that the awareness of image manipulations was reflected in subsequent eye-movements. However, our current results suggest that, in fact, overt visual attention preceded the awareness of the stimulus manipulation.

Our results extend recent experimental and theoretical evidence pointing into the direction of neurally distinct mechanisms for visual awareness and attention [3], [26], [27], [28]. For instance, using faint stimuli that reached perceptual awareness in only about 50% of the trials, Wyart et al. (2008) showed that visual awareness correlated with an increase in mid-frequency gamma-band activity at the contralateral visual cortex, whereas covert visual attention modulated high-frequency gamma-band activity in the same region. These results suggest that the neural correlates of the two processes are in fact distinct. In addition to this, there is electrophysiological evidence suggesting that processes of attentional selection precede visual awareness. Fernandez-Duque et al. [4] investigated event related potentials (ERPs) related to visual attention and aware vs. unaware changes in a flicker paradigm [29]. Their data was grouped based on the subjects' awareness of changes, either aware or unaware, in subsequently presented scenes. The results showed early, attention-related components over frontal and parietal sites, followed by a late component that was related to awareness of visual change. The latter component exhibited distinct topography, by being broadly distributed with its center in medial centro-parietal regions. The described attentional regions broadly correspond to earlier results of Beck et al. [30] and also of Huettel et al. [31]. Comparing fMRI responses in a similar flicker paradigm, they associated the awareness of change with enhanced BOLD responses in parietal and right dorsolateral prefrontal cortex as well as extrastriate visual cortex. Similarly, the data presented in [32], [33], [34] suggests based on behavioral and electrophysiological measurements that attention and consciousness are initially independent, whereas later, higher-level visual awareness is strongly depended on focused attention. Taken together, there is converging evidence for the view that visual awareness and visual attention rely on two distinct neural mechanisms and that patterns of activity correlated with visual attention precede effect of visual awareness. Our results now clarify the interaction of both phenomena on a behavioral level by showing that patterns of overt visual attention have a causal impact on the resulting object awareness.

Converging evidence for our results comes from previous studies investigating the effects of eye-movements on perceptual illusions [35], [36], [37] and on perceptual oscillations [2], [6], [22], [38]. For the latter, informed subjects and prolonged stimulus presentations were used in order to induce regular perceptual oscillations. Although this approach has the clear advantage of comparably easy data collection, it severely complicates an analysis of the direction of causality, as all recorded eye-movements precede but also follow perceptual events. Also, in addition to being a rather artificial setting, the study of perceptual oscillations has the problem that the data is collected from non-naive subjects that become ‘stimulus-experts’ due to the long presentation time and because they are typically presented with both interpretations prior to the experimental trial. In the current set of experiments, we overcome these limitations by only analyzing data recorded prior to the initial perception of the object's identity and by excluding all subjects with prior knowledge of the stimuli.

The most important difference of our approach is that we investigate overt visual attention occurring prior to the first conscious perception of the subjects (perceptual formation) whereas the data recorded from perceptual oscillations is always accompanied with active perceptual interpretations and perceptual switches. The same argument holds for the previously reported results of perceptual events on pupil dilation, which were always based on the recordings of perceptual switches [12]. Because of this, it was previously unclear whether the neuronal mechanisms of pupil dilation involving norepinephrine release (see below) followed or lead to the perceptual switch. In the current experiments, we show pupil dilation effects based on the initial perceptual interpretation following a time in which the subjects were not yet aware of any object identity. With regard to the underlying mechanism, Einhäuser et al. [12] argue that pupil dilation recorded around perceptual switches reflects norepinephrine release from locus coeruleus (LC). LC has been implicated in regulating the balance between exploitation and exploration within the sensory domain and to consolidate perceptual decisions [39], [40]. This exploitation-exploration model is very well in line with our results.

Similar to our disambiguated stimuli, albeit again based on data recorded from perceptual oscillations, Pomplun et al. [22] showed that changes of ambiguous stimuli can result in perceptual biases, leading the subjects to perceive one interpretation significantly more often than the other. Kawabata and Mori [6] provided evidence in line with the results of experiment 2 by showing that the perceptual outcome of the subjects can be altered if forced onto one stimulus position. In our case, however, the experimental manipulation is much more subtle, because the attended position is only altered prior to the actual stimulus presentation and not during the complete trial.

An open research question is on what basis the targets of eye-movements are selected during the initial phase in which the object is not yet recognized. Possible mechanisms include bottom-up visual salience (either mediated via low-level features and feature-combinations represented in V1 [41], [42], [43] or determined by a saliency-based approach combining multiple feature maps [5], [44]) and high-level, hypothesis driven attention working in a top-down manner [45], [46]. In either case, it might be of special importance to differentiate local stimulus properties from the effects of stimulus context. The gist of a scene can provide a strong cue for the object identity and is therefore a promising candidate for future research in this direction. Please note that the current findings do not argue against the task-dependent view of overt visual attention [46], [47], because attention can be guided towards task-relevant objects without requiring constant and conscious awareness of their identities. Our results are compatible with a constructive view of perception [48] and provide new evidence for the impact of eye movements during the formation object awareness [49].

## Supporting Information

Figure S1 **Stimuli.** Shown are the ten ambiguous and disambiguated stimuli that were used for the analysis. The first column contains the ambiguous image, the second and third the respective disambiguated versions.
(TIF)

Figure S2 **Experiment 2.** Shown is an example stimulus together with the calculated centroids of the 80% congruency regions (circles), as marked by a set of independent subjects. The colored crosses correspond to the shifted fixation cross positions used in experiment 1, the black cross shows the centered fixation cross used in experiment 1.
(TIF)

Figure S3 **Pupil Size Analysis.** The averaged pupil size z-scores from the (**a**) percept formation condition (data from experiment 1) and (**b**) the control experiment in which subjects pressed the same keyboard button whenever they wished to do so. The shaded area around the pupil diameter shows the SEM. Time periods with a significant positive slope are marked with a light grey bar. (**c**) A statistical comparison of the perceptual- and motor-task showing significant differences at 528 ms before the button press.
(TIF)

Analyses S1 **The subject's pupil dilation was used as additional marker of the perceptual decision.** To better estimate the time-point of the perceptual decision, we contrast pupil dilation changes preceding perceptual and motor-decisions. This revealed significant differences from 528 ms before to 3000 ms after the button press.
(DOCX)
