# Supplementary figures and images for: Overt Visual Attention as a Causal Factor of Perceptual Awareness
Source: PLoS One. 2011 Jul 25;6(7):e22614. doi: 10.1371/journal.pone.0022614 (PMC3143177; doi:10.1371/journal.pone.0022614)

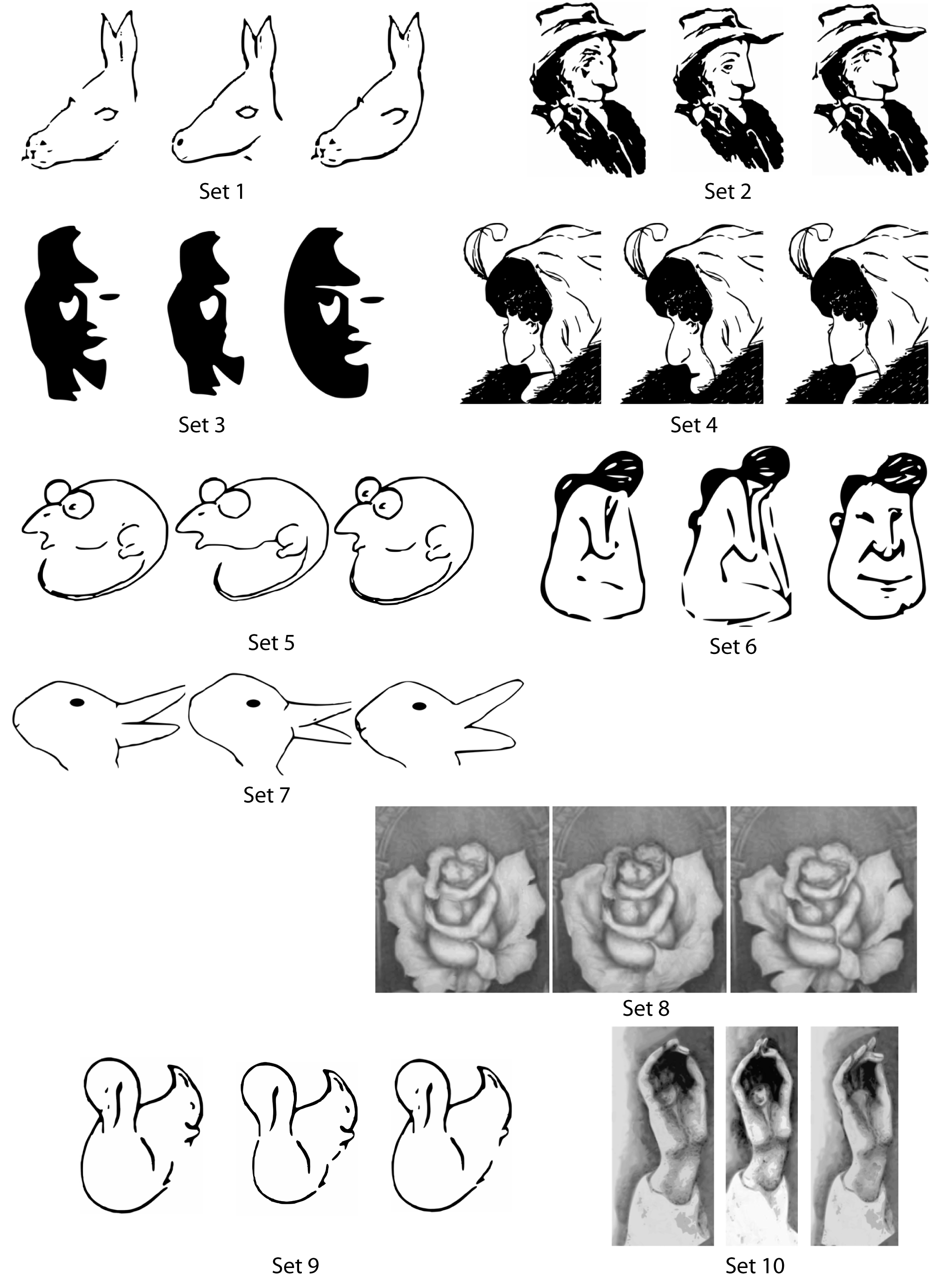

Supplement: Figure S1 — Stimuli. Shown are the ten ambiguous and disambiguated stimuli that were used for the analysis. The first column contains the ambiguous image, the second and third the respective disambiguated versions. (TIF) [file pone.0022614.s001.tif]

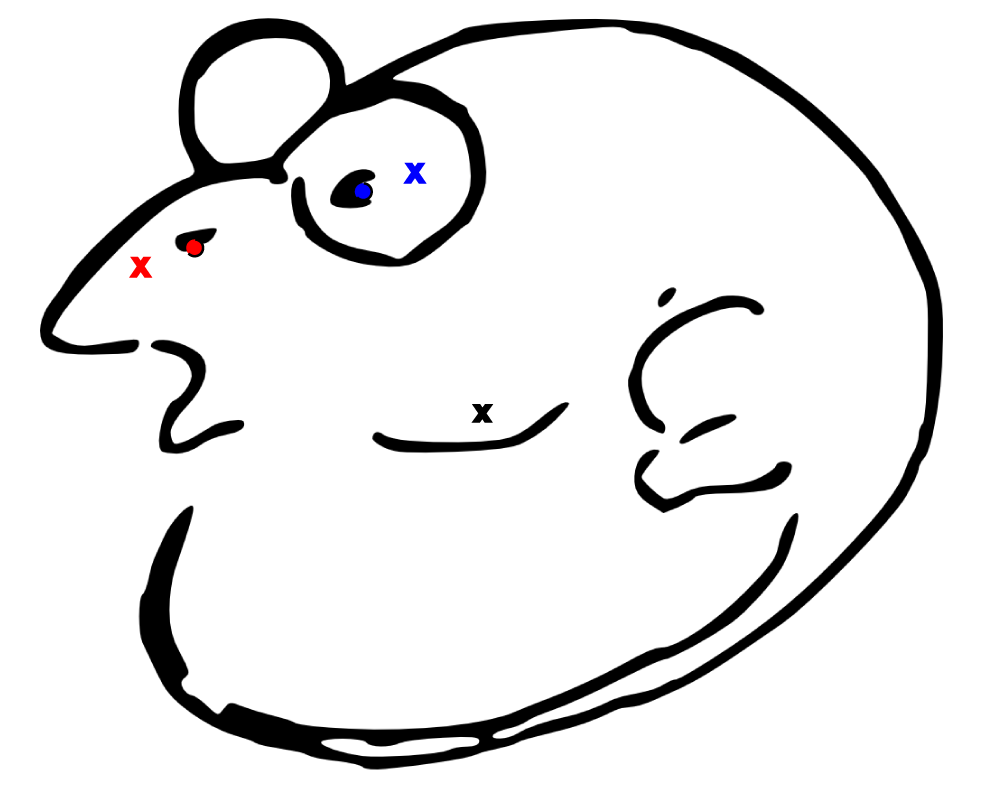

Supplement: Figure S2 — Experiment 2. Shown is an example stimulus together with the calculated centroids of the 80% congruency regions (circles), as marked by a set of independent subjects. The colored crosses correspond to the shifted fixation cross positions used in experiment 1, the black cross shows the centered fixation cross used in experiment 1. (TIF) [file pone.0022614.s002.tif]

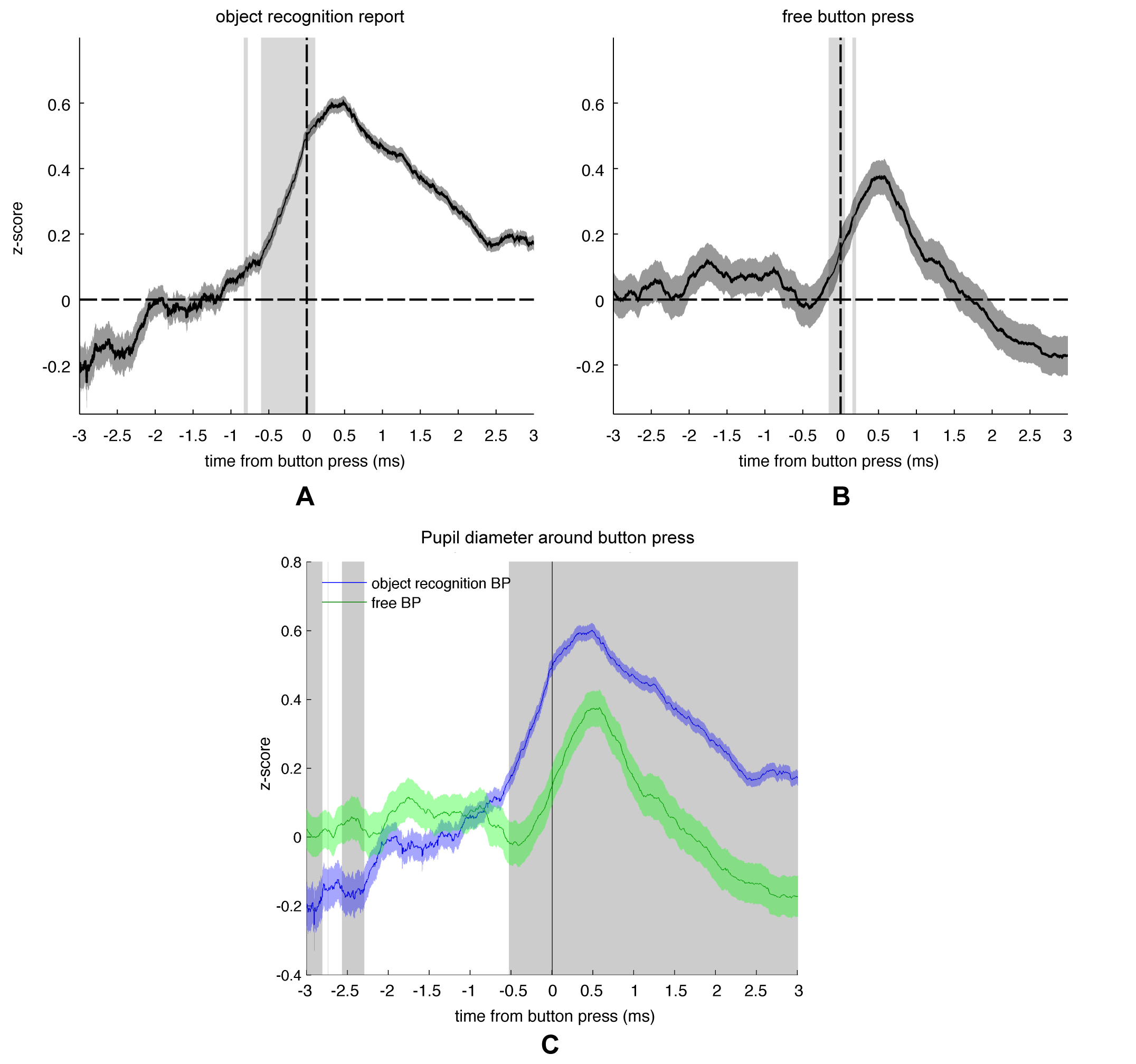

Supplement: Figure S3 — Pupil Size Analysis. The averaged pupil size z-scores from the (a) percept formation condition (data from experiment 1) and (b) the control experiment in which subjects pressed the same keyboard button whenever they wished to do so. The shaded area around the pupil diameter shows the SEM. Time periods with a significant positive slope are marked with a light grey bar. (c) A statistical comparison of the perceptual- and motor-task showing significant differences at 528 ms before the button press. (TIF) [file pone.0022614.s003.tif]
