# Supplementary material for: Overt Visual Attention as a Causal Factor of Perceptual Awareness
Source: PLoS One. 2011 Jul 25;6(7):e22614. doi: 10.1371/journal.pone.0022614 (PMC3143177; doi:10.1371/journal.pone.0022614)
Supplement: Analyses S1 — The subject's pupil dilation was used as additional marker of the perceptual decision. To better estimate the time-point of the perceptual decision, we contrast pupil dilation changes preceding perceptual and motor-decisions. This revealed significant differences from 528 ms before to 3000 ms after the button press. (DOCX) [file pone.0022614.s004.docx]

**Supplementary Analyses**

**Pupil Analysis.** The diameter of the pupil was previously shown to increase at the time of cognitive events [1], as for instance switches in perception [2, 3]. However, it has been argued that part of the pupil response is due to response execution [1, 3] and therefore not related to perception per se. It is argued that especially the peak of the pupil dilation is modulated by this effect. Here, we are interested in the onset of the pupil dilation in relation to the report of conscious recognition of the object identity. Compared to the button press, which of course includes the time required for the motor response, the start of pupil dilation is a better estimate of the completed recognition process because it is either related to the perceptual event or to the response preparation signaling object awareness. Both processes are closer to the time point of object recognition than a pure button press.

For the current pupil analysis, the data from experiment 1 was related to data collected in a control task (conducted directly after experiment 2). Subjects were presented 6 pink noise images and 2 blank screens, resulting in a total of 8 trials. The subjects’ task was to press a button as often and whenever they wanted. They were allowed to freely move their eyes during this task. Since the stimuli do not depict any object or meaningful structure, these trials did not include any object awareness, but only a self paced motor preparation and response. To assess differences in perceptual and motor-related processes, the pupil size around these simple motor actions were then compared to the pupil size around motor report of percept formation in experiment 1.

Pupil size was recorded by the Eyelink II system with 500 Hz. In the original experiment, pupil traces from all three blocks of experiment 1 were extracted from stimulation onset to 10 seconds after onset. All trials interrupted by the experimenter and trials in which the button press occurred earlier than 500 ms or later than 10 seconds after stimulus onset were discarded. Additionally, all trials in which the interval between two button presses was smaller than 500 ms were removed. Pupil traces were trial wise z-transformed, periods of 100ms before and after automatically detected blinks were excluded together with manually detected artifacts. The removed data were replaced via linear interpolation. The traces were then aligned to the button press and data within +/- 3s around the button press were averaged over all trials (n=790).

From the control task, only the last button presses within each trial were considered to allow for maximal delay to the last perceptual change, i.e. the switch from drift correction to pink noise or blank screen. Identical to the experimental data, pupil responses were z-transformed and artifacts were removed. As before, pupil traces from the time window of +/- 3s around the last button press were extracted, aligned to the button press and averaged (n=147).

Pupil diameter is plotted for both conditions in s*upplementary figure S3*. First, we compared the pupil size maxima of individual trials in the two conditions and found that they were significantly higher in the object recognition condition (mean=0.6) as compared to the control condition (mean=0.38; Wilcoxon’s rank sum test; p<0.001).

In order to estimate the point in time at which the two curves significantly deviate from each other, we tested the pupil diameter traces at each 2ms time samples within the 3-second period around the button press. The analysis revealed a significantly larger pupil diameter in the recognition condition from 528 ms prior to 3000 ms after to the button press (t-tests, p<0.05 FDR corrected; with Satterthwaite's approximation for unequal variances).

These results show that the human pupil reacts to motor actions such as button presses in the absence of visual stimulation. However, if the motor action follows a perceptual event, such as object recognition of line drawings, the pupil dilation is facilitated. This additive pupil reaction to percept formation is in line with previous results from experiments investigating perceptual switches on similar stimuli [2, 3]. On an aggregated level, the pupil dilation distinguishes between isolated button presses and button presses after object recognition. Since the object recognition occurs before the button press and contributes to the size of the pupil dilation, the onset of the pupil dilation can be used as a measure of the on average completed object recognition.

1. Richer F, Silverman C, Beatty J (1983) Response Selection and Initiation in Speeded Reactions: A Pupillometric Analysis. Journal of Experimental Psychology 9:360-370.
2. Einhäuser W, Stout J, Koch C, Carter O (2008) Pupil dilation reflects perceptual selection and predicts subsequent stability in perceptual rivalry. Proc Natl Acad Sci U S A 105:1704--1709.
3. Hupé JM, Lamirel C, Lorenceau J (2009) Pupil dynamics during bistable motion perception. Journal of Vision 9:1--19.
